# Dataset on marine ecosystem services supplied by coral reefs, sandy beaches and coastal lagoons in different eutrophication states

**DOI:** 10.1016/j.dib.2019.104078

**Published:** 2019-05-29

**Authors:** Charlène Kermagoret, Joachim Claudet, Valérie Derolez, Maggy M. Nugues, Vincent Ouisse, Nolwenn Quillien, Denis Bailly

**Affiliations:** a Univ Brest, Ifremer, CNRS, UMR 6308, AMURE, IUEM, 29280, Plouzane, France; b Département des Sciences Naturelles, Institut des Sciences de la Forêt Tempérée, Université du Québec en Outaouais, Gatineau, Canada; c National Center for Scientific Research, PSL Université Paris, CRIOBE, USR 3278 CNRS-EPHE-UPVD, Maison des Océans, 195 rue Saint-Jacques 75005 Paris, France; d Labex Corail, CRIOBE, 98729 Moorea, French Polynesia; e MARBEC, Ifremer, IRD, Univ Montpellier, CNRS, Av. Jean Monnet, 30171 - 34203 Sète Cedex, CS, France; f EPHE, PSL Research University, UPVD-CNRS, USR3278 CRIOBE, F-66860 Perpignan, France; g France Energies Marines, 29200 Brest, France

**Keywords:** Ecosystem services, Marine ecosystems, Eutrophication, Marine biodiversity

## Abstract

This data article provides indicators of Ecosystem Service (ES) supply for coral reefs, sandy beaches and coastal lagoons in different ecological states regarding eutrophication. 14 ES are considered: food through fisheries; material; molecules; coastal protection; nutrient regulation; pathogen regulation; climate regulation; support of recreational and leisure activities; contribution to a pleasant landscape; contribution to culture and territorial identity; emblematic biodiversity; habitat; trophic networks; recruitment. For each ecosystem 3 to 4 eutrophication states are described. Indicators of ES supply are filled on the basis of a literature review supplemented with expert-knowledge. A semi-quantification of the indicator value is finally provided. Tendencies and trade-offs between ES are analyzed in How does eutrophication impact bundles of ecosystem services in multiple coastal habitats using state-and-transition models [1].

**Table:** Specifications Table [please fill in right-hand column of the table below]

Subject area	*Environmental management*
More specific subject area	*Ecosystem services and biodiversity*
Type of data	*Table*
How data was acquired	*Literature reviewand expert-knowledge coded on Microsoft Excel 2016*
Data format	*Raw*
Experimental factors	*For each ecosystem, 3 to 4 eutrophication states are considered and described by supply indicators of 14 ES*
Experimental features	*Eutrophication states are described by dominant species and associated biodiversity. Supply indicators of ES are qualitative or quantitative information, depending on available data. Information is then coded to assess the variation of ES supply between eutrophication states (five levels from very low to very high).*
Data source location	*The literature review encompassed knowledge obtained and disseminated on a global scale, while expert knowledge focused on data observed on a more local scale, based on their field studies. However, experts had a good understanding of these ecosystems that allowed them to pronounce in a qualitative way where data gaps were identified.*
Data accessibility	*Data are provided in this article*
Related research article	C. Kermagoret, J. Claudet, V. Derolez, M. Nugues, V. Ouisse, N. Quillien, Y. Baulaz, P. Le Mao, P. Scemama, D. Vaschalde, D. Bailly, R. Mongruel, Comparison of how eutrophication affects bundles of ecosystem services in multiple coastal habitats using state-and-transition models, Ocean Coast. Manage. 174 (2019) 144–153. https://doi.org/10.1016/j.ocecoaman.2019.03.028

**Table:** 

**Value of the data** • The dataset is useful for understanding the relations between ecological functions, ecological states and ES. • Scientific can benefit from this data to carry out ES assessment or to inform decision-making processes and management strategies regarding eutrophication. • Practitioners and policy-makers can benefit from this data to adopt management strategies or measures for biodiversity conservation. • The data provide a first overview of the effects of eutrophication on ES bundles and can serve as a basis for a larger database. • The data can be used for further insight the trade-offs and synergies between ES and can be used for modelling. • The dataset helps to build relationships between disparate data on the effect of eutrophication on ecological function and ES.

## 1 Data

The dataset contains qualitative description of ES provided by coral reefs, sandy beaches and coastal lagoons in different eutrophication states. Three to four eutrophication states are considered, from non-eutrophic to hyper-eutrophic state. 14 ES are considered: food through fisheries; material; molecules; coastal protection; nutrient regulation; pathogen regulation; climate regulation; support of recreational and leisure activities; contribution to a pleasant landscape; contribution to culture and territorial identity; emblematic biodiversity; habitat; trophic networks; recruitment. The capacity of the ecosystem to provide ES is described for each ES in each eutrophication state using qualitative information collected in the literature completed by expert-knowledge. Data are presented in double entry matrix (ES/eutrophication states) for coral reefs (Table 1), sandy beaches (Table 2), coastal lagoons (Table 3). These capacities - or supplies - are finally semi-quantified in order to compare them in the bundle.

**Table 1:** Indicators and semi-quantification (SQ) of ES supply for coral reefs in 3 eutrophication states ("0: inexistent", "1: very low", "2: low", "3: medium", "4: high", "5: very high").

Eutrophication states	Non-eutrophic Dominance of hard coral		Eutrophic Dominance of algal turf and macroalgae		Hyper-eutrophic Dominance of cyanobacterial mats	
Human food through fisheries	Coral-dominated reefs are the most productive [12]	5	Depends on structural complexity. Multi-species fisheries can shift towards herbivorous fish species dominated yields [13]	3	Bottoms lacking structural complexity become very poor in target species.	1
Material (organism for domestic uses, industry, agriculture …)	Ornamental purposes	2	Ornamental purposes	1	Ornamental purposes	1
Molecules (organisms from which are extracted molecules potentially useful for medicine)	Living coral reef supports a high diversity of organisms and thus potential biomolecules	4	Lower diversity of organisms and thus lower potential for biomolecules	2	Cyanobacteria and sponges are chemically rich and can be used in medical chemistry, pharmacology or phytopharmacy.^a^	4
Coastal protection (vegetal or animal reef supplying a protection against erosion and submersion)	Coral reef can dissipate 97% of the wave energy that would otherwise impact shorelines [15]	5	The loss of corals, the increased water depth between the reef crest and the surface should result in a less effective ES.	1	The loss of corals, the increased water depth between the reef crest and the surface should result in a nonexistent ES.	0
Nutrient regulation (ecosystem capacity to supply a "good quality water", limiting the risk of eutrophication, encouraging shellfish farming…)	Intensively performed by zooxanthellae in living coral reef.	5	The risk of hyper-eutrophication is greater in altered or dead coral reefs.	3	Cyanobacterial mats and sponges, able to provide the ES, can be easily washed away by storms.	1
Pathogen regulation (ability of ecosystems to purify the environment through hyperfiltration processes)	High capacity of pathogen regulation thanks coral microbiome.	4	Macroalgae alter the coral microbiome and elevate putative pathogen loads [16], [17]	2	Macroalgae alter the coral microbiome and elevate putative pathogen loads [16], [17]	1
Climate regulation (GES storage or sequestration)	Living coral reefs widely contribute to climate regulation, stocking GES through the production of carbonates [18]	4	The loss of corals results in a less effective ES.	2	The loss of corals results in a less effective, or even nonexistent, ES.	1
Support of recreational and leisure activities	In many tropical societies, relations to nature are often very different from those related to the Western lifestyle and the distinction between culture and nature is sometimes blurred.	5	In these tropical contexts, the difficult resilience and adaptive capacity to abrupt changes in coral reefs (eutrophication and other pressures) can alter cultural ES [19] Emblematic biodiversity also decreases.	3	In these tropical contexts, the difficult resilience and adaptive capacity to abrupt changes in coral reefs (eutrophication and other pressures) can alter cultural ES [19] Emblematic biodiversity also decreases.	1
Contribution to a pleasant landscape		5		3		1
Contribution to culture and territorial identity		5		2		1
Emblematic biodiversity (i.e. protected or rared species)		5		2		0
Habitat (nursery, reproduction area…)	Corals provide shelter and food for a large diversity of benthic organisms and allow the creation of complex trophic networks. The three dimensional structure of corals are important to fish recruitment, which can, in turn, increase herbivory and favor coral dominance via positive feedback mechanisms [20]	5	Algal-dominated state can benefit some herbivorous fishes, but large fleshy macroalgae are often unpalatable to fishes. Mesopredators can switch prey, shortening food chains, in response to coral reef degradation [21]	3	Cyanobacterial mats are often unpalatable to fishes. Mesopredators can switch prey, shortening food chains, in response to coral reef degradation [21]	1
Trophic networks		5		3		1
Recruitment		5		3		1

a However, chemical defense could lessen in absence of consumers. For example, sponge communities have become dominated by fast-growing species that lack chemical defenses on reefs where sponge-eating angelfishes and parrotfishes have been removed by overfishing [14].

**Table 2:** Indicators and semi-quantification (SQ) of ES supply for sandy beaches in 3 eutrophication states ("0: inexistent", "1: very low", "2: low", "3: medium", "4: high", "5: very high").

Eutrophication states	Non-eutrophic Reference species (Tellinidae, Spionidae, Amphiuridae, Nephtyidae)		Eutrophic Green algae proliferation (Donacidae, Oweniidae, Magelonidae, Nephtyidae)		Hyper-eutrophic Green tide (Donacidae, Oweniidae)	
Human food through fisheries	Sandy beaches support professionnal fisheries of the bivalve *Donax trunculus*, which is of commercial importance [22] They are also a high spot for surf casting targeting Moronidae (*Dicentrarchus labrax*).	5	A decrease in *Donax trunculus* density has been shown [7]. However, the presence of floating mats (within 0.5-1.5m of water) seemed to form the perfect hunting ground for the seabass (pers. obs.).	3	At strong eutrophication state, a decrease in *Donax trunculus* density has been shown [7]	1
Material (organisms for domestic uses, industry, agriculture …)	Driftwood and seashell for ornamental purposes	1	Driftwood and seashell for ornamental purposes	1	Driftwood and seashell for ornamental purposes	1
Molecules (organisms from which are extracted molecules potentially useful for medicine)	The polychaete *Arenicola marina* is collected and bred to get hemoglobin for medical uses. As the diversity that is harboured by sandy beaches is high, specilized and unique, there are potentially lots of useful molecules.	3	*Arenicola marina* is collected and bred to get hemoglobin for medical uses.	3	*Arenicola marina* can be impacted by green tides.	2
Coastal protection (vegetal or animal reef supplying a protection against erosion and submersion)	Specific fauna and flora (mainly bioturbating organisms) able to reduce the hydrodynamics and stabilize the substrate.	3	Ulva mats impact the hydrodynamics [23] thus affecting the sediment transport between subtidal sands, sandy shores and dunes and ultimately the function of protection	1	Ulva mats impacts the hydrodynamics [23] thus affecting the sediment transport between subtidal sands, sandy shores and dunes and ultimately the function of protection	1
Nutrient regulation (ecosystem capacity to supply a "good quality water", limiting the risk of eutrophication, encouraging shellfish farming…)	Beach ecosystems are important in processing large quantities of organic material and recycling nutrients back to coastal waters [24]	4	The release in excess of nutrients from lands and the presence of heterogeneous cover of green macroalgae probably affect the filtering function of the system.	3	The release in excess of nutrients from lands and the presence of heterogeneous cover of green macroalgae probably affect the filtering function of the system.	1
Pathogen regulation (ability of ecosystems to purify the environment through hyperfiltration processes)	Specific literature does not exist but as sandy beaches are made of porous sands that form an excellent "digestive and incubating system" [24], they most probably filter and purify the environment	4		3		1
Climate regulation (GES storage or sequestration)	Autotroph systems [25]Sequestration through the phytoplanktonic and microphytobenthic activity (short term).	3	Probably short term C sequestration through the photosynthetic activity of green algae, phytoplankton and microphytobenthos	3	Probably short term C sequestration mainly through the photosynthetic activity of green algae	3
Support of recreational and leisure activities	Landscape, leisure activities (e.g. go to the beach), territorial activities (e.g. recreational fisheries) and emblematic biodiversity (avifauna) are important [22], [24]	5	Landscape, leisure activities, territorial activities and emblematic biodiversity are impacted by green algae [22], [24], [26]	4	Landscape, leisure activities, territorial activities and emblematic biodiversity are impacted by green tides [22], [24], [26]	0
Contribution to a pleasant landscape		5		3		0
Contribution to culture and territorial identity		5		3		0
Emblematic biodiversity (i.e. protected or rared species)		5		4		2
Habitat (nursery, reproduction area…)	Nursery function [22]	5	Eutrophication impacts nursery function [27]	3	Eutrophication impacts nursery function [27], [28]	1
Trophic networks	The food web is complex, showing several potential carbon pathways and diverse trophic niches [27]	5	The trophic network is in process of homogenization (27]	3	The trophic network is homogenized/simplified and shows less niche differentiation [27]	1 2
Recruitment	High recruitement for flatfish and other species [29]	4	Ulva mats influence local hydrodynamics, which in turn influence the recruitment of some species. In Brittany, the heterogenous cover of Ulva enhances the recruitment of *Donax vitattus*[7]	5	When the Ulva biomass is too high, macroalgae affect the recruitment, community structure and production of benthic fauna, including meiofauna, macrofauna and flatfish [7]	2

**Table 3:** Indicators and semi-quantification (SQ) of ES supply for coastal lagoons in 4 eutrophication states ("0: inexistent", "1: very low", "2: low", "3: medium", "4: high", "5: very high").

Eutrophication states	Non-eutrophic		Eutrophic		Eutrophic		Hyper-eutrophic	
Human food through fisheries	Carrying capacity for shellfish farming of oligotrophic lagoons is questionned.	4	Carrying capacity for shellfish farming of slightly eutrophic lagoons is better.	5	Impact of anoxic crises on shellfish stocks (death).	4	Impact of anoxic crises on shellfish stocks (death).	4
Material (organisms for domestic uses, industry, agriculture …)	NA	0	NA	0	NA	0	NA	0
Molecules (organisms from which are extracted molecules potentially useful for medicine)	Potential	1	Potential	1	Potential	1	Potential	1
Coastal protection (vegetal or animal reef supplying a protection against erosion and submersion)	Seagrass meadows have the capacity to attenuate waves and to slow down currents [30]	3	Decrease with the alteration and decline of seagrass meadows [30]	2	Decrease with the alteration and decline of seagrass meadows [30]	1	Decrease with the alteration and decline of seagrass meadows [30]	1
Nutrient regulation (ecosystem capacity to supply a "good quality water", limiting the risk of eutrophication, encouraging shellfish farming…)	Seagrass beds play an important role in regulating benthic nutrient fluxes in lagoons as they increase the ability to store nutrients sustainably.	5	The flow of nutrients from the sediment to the water column and, at the same time, eutrophication levels are thus greater in lagoons without seagrass [31], [32]	3	Eutrophication levels are greater in lagoons without seagrass [31], [32]	2	Eutrophication levels are greater in lagoons without seagrass [31], [32]	1
Pathogen regulation (ability of ecosystems to purify the environment through hyperfiltration processes)	Algicidal effects of *Zostera marina L.* and *Zostera noltii Hornem.* on *Alexandrium catenella*[33] Seagrass ecosystems reduce exposure to bacterial pathogens [34] but oligotrophication can lead to emergence of toxic dinoflagellate [35]	3	Algicidal effects of Zostera marina L. and Zostera noltii Hornem. on Alexandrium catenella [33]	4	A decrease of seagrass leads to a stronger exposure to bacterial pathogens of humans, fishes, and invertebrates [34]	2	A decrease of seagrass leads to a stronger exposure to bacterial pathogens of humans, fishes, and invertebrates [34]	1
Climate regulation (GES storage or sequestration)	Potential long-term sequestration in the sediment through perennial macrophytes	5	A decrease of perennial macrophytes leads to reduce the sequestration in the sediment	4	A decrease of perennial macrophytes leads to reduce the sequestration in the sediment	2	A decrease of perennial macrophytes leads to reduce the sequestration in the sediment	1
Support of recreational and leisure activities	Scuba-diving, snorkling, sailing, wind-surf	4	Scuba-diving, snorkling, sailing, wind-surf	4	Avifauna observation	3	Avifauna observation	3
Contribution to a pleasant landscape	Avifauna, and elements of underwater seascape [36]	5	Avifauna, and elements of underwater seascape [36]	4	Avifauna	2	Avifauna	2
Contribution to culture and territorial identity	Biodiversity of coastal lagoons are a socialization area, sometimes assimilated to an urban park [36]	4	Socialization area [36]	4	Socialization area [36]	4	Socialization area (36]	4
Emblematic biodiversity (i.e. protected or rared species)	Protected and rare species (*Zostera sp., Hippocampus sp.*, avifauna)	4	Protected and rare species (*Zostera sp., Hippocampus sp.*, avifauna)	4	Avifauna (flamingos) [37]	3	Avifauna (flamingos) [37]	3
Habitat (nursery, reproduction area…)	Coastal lagoons provide higher temperature during growth and food to *Sparus aurata*, which allow good lipid reserves, and large sizes of juveniles, which may be important to their survival over winter [38] Zostera meadows are a habitat for many species.	5	A decrease of Zostera meadows impacts the ES	4	A decrease of Zostera meadows impacts the ES	4	Coastal laoons provide higher temperature during growth and food to Sparus aurata, which allow good lipid reserves, and large sizes of juveniles, which may be very important to their survival over winter [38]	5
Trophic networks	High complexity [39]	5	A decrease of Zostera meadows leads to less complexity	4	A decrease of Zostera meadows leads to less complexity	2	A decrease of Zostera meadows leads to less complexity	1
Recruitment	Carrying capacity for juvenile oysters of oligotrophic lagoons is questioned [40]	3	Capacity for juvenile oysters	4	Capacity for juvenile oysters	4	Capacity for juvenile oysters	4

## 2 Experimental design, materials and methods

For each ecosystem, eutrophication states are described by dominant species and associated biodiversity. First eutrophication state of coral reefs is characterized by the dominance of hard corals [1]. Other benthic communities present are algal turfs, crustose coralline algae, sponges and benthic cyanobacterial mats [2]. The increasing pressure leads to the development of algal turfs and fleshy macroalgae which are fast-growing organisms and a gradual decline of coral cover [3], in particular from competitive losses against algae under conditions of reduced herbivory [4]. With a continuous and increasing pressure, benthic cyanobacterial mats increase and become dominant at the expense of algal turfs and macroalgae while sponges showed a more limited but significant increase. Benthic cyanobacteria mats benefit from increased levels of nutrient [5] but also from high grazing pressure and elevated water temperature [6].

First eutrophication state of sandy beaches is described by groups of species defined as reference species living in a non-eutrophic ecosystem, where no green tides occur [1]. In some French Atlantic sandy beaches, these reference species, for marine benthic macrofauna, are part of Tellinidae, Spionidae, Amphiuridae and Nephtyidae families [7]. The continuous supply of nutrients (exogenous inputs or release from sediments) causes a slight excess and leads to the gradual development of green algae. As a response, dominant species change in this eutrophic ecosystem with an appearance of new dominant species (Donacidae, Oweniidae, Magelonidae) and the decrease, even the disappearance, of some reference species (Tellinidae, Spionidae, Amphiuridae) [7]. Where hydrodynamic conditions are favorable, the massive supply of nutrients leads to the massive and rapid development of green algae forming green tides. Species of reference have disappeared in favor of species (Donacidae, Oweniidae) better adapted to eutrophic conditions.

First eutrophication state of primary production of coastal lagoon is characterized by a dominance of reference species that are typical of a lagoon environment in oligotrophic conditions [1]. For French Mediterranean coastal lagoons, the reference genus are the marine phanerogams Zostera and Ruppia which form seagrass beds, and perennial benthic macroalgae (eg. Cystoseira sp., Acetabularia sp.) [8], [9]. The continuous supply of nutrients causes a slight excess and leads to the gradual disappearance of the reference species and the slow and sustainable development of algae [10]. The second state is dominated by a dominance of opportunistic and epiphytic macroalgae. The massive supply of nutrients leads to the massive and rapid dominance of free-floating blooming opportunistic algae. In the most eutrophicated systems, phytoplankton community dominates the water column.

We used the classification of the Common International Classification of Ecosystem Services (CICES) and the list of marine ES defined by Liquete et al. [11] to defined the ES constituting bundles. The main distinction between these classifications concerns supporting services or ecological functions. These latter are the underpinning structures and processes that ultimately give rise to ecosystem services - sometimes defined as ‘intermediate services’. They are not covered in CICES which seeks to only identify the final services that link to the goods and benefits that are valued by people (i.e. demand). Since we focus here on the ES supply, main ecological functions are considered as recommended by Liquete et al. [11].

Each step involved a literature review regarding ecosystem responses to eutrophication that was supplemented with expert-knowledge. The literature review encompassed knowledge obtained and disseminated on a global scale, while expert knowledge focused on data observed on a more local scale, based on their field studies. However, experts had a good understanding of these ecosystems which allowed them to pronounce in a qualitative way where data gaps were identified. All information were compiled within Table 1. Information was then coded to summarize the variation of ES supply between states. Five levels of ES supplied were considered: "0: inexistent", "1: very low", "2: low", "3: medium", "4: high", "5: very high".

## Conflict of Interest

The authors declare that they have no known competing financial interests or personal relationships that could have appeared to influence the work reported in this paper.
